# Effect of cardiolipin on the antimicrobial activity of a new amphiphilic aminoglycoside derivative on *Pseudomonas aeruginosa*

**DOI:** 10.1371/journal.pone.0201752

**Published:** 2018-08-20

**Authors:** Jitendriya Swain, Micheline El Khoury, Julie Kempf, Florian Briée, Patrick Van Der Smissen, Jean-Luc Décout, Marie-Paule Mingeot-Leclercq

**Affiliations:** 1 Pharmacologie Cellulaire et Moléculaire, Louvain Drug Research Institute, Université catholique de Louvain, Brussels, Belgium; 2 Département de Pharmacochimie Moléculaire, Université Grenoble Alpes, CNRS, Grenoble, France; 3 de Duve Institute, Université catholique de Louvain, Brussels, Belgium; University of Cambridge, UNITED KINGDOM

## Abstract

Amphiphilic aminoglycoside derivatives are promising new antibacterials active against Gram-negative bacteria such as *Pseudomonas aeruginosa*, including colistin resistant strains. In this study, we demonstrated that addition of cardiolipin to the culture medium delayed growth of *P*. *aeruginosa*, favored asymmetrical growth and enhanced the efficiency of a new amphiphilic aminoglycoside derivative, the 3’,6-dinonylneamine. By using membrane models mimicking *P*. *aeruginosa* plasma membrane composition (POPE:POPG:CL), we demonstrated the ability of 3’6-dinonylneamine to induce changes in the biophysical properties of membrane model lipid systems in a cardiolipin dependent manner. These changes include an increased membrane permeability associated with a reduced hydration and a decreased ability of membrane to mix and fuse as shown by monitoring calcein release, Generalized Polarization of Laurdan and fluorescence dequenching of octadecyl rhodamine B, respectively. Altogether, results shed light on how cardiolipin may be critical for improving antibacterial action of new amphiphilic aminoglycoside derivatives.

## Introduction

Bacterial resistance to all classes of antibiotics, including aminoglycosides, is becoming a global public health crisis [1] requiring urgent actions. Besides measures to encourage the appropriate use of antibiotics, discovery and development of new active molecules are urgently required. Driven by the unique architecture of bacterial envelopes and biophysical properties of bacterial cell membranes, the search for new membrane-active antibacterials is promising [2].

Membrane-active antibiotics target essential and preserved structures among various bacterial species. More and more they are described as chemosensitizer to increase the activity of other antibiotics [3]. They have also been developed for their potential alternatives to the resistance-prone conventional antibiotics and for their activity against slow-growing or dormant bacteria as well as on biofilms.

At the molecular level, lipids like cardiolipin play a critical role in bacterial physiology, especially for cytokinesis [4;5] and activity of the respiratory complex [6]. Cardiolipin is a dimeric anionic phospholipid (CL; S1 Fig) with unique biochemical and biophysical properties [7]. In Gram-negative bacteria the content of cardiolipin is found between 5−30% and is dependent upon bacterial strains and their state of growth [4;5]. The level of cardiolipin can be modulated by environmental factors [8–10]. Cardiolipin is accumulated at the cell poles and septum of rod-shaped bacteria (i.e. *Pseudomonas aeruginosa*) ensuring proper spatial segregation, recruitment and/or activity of membrane proteins [11;12]. Cardiolipin also specifically induces an increase in protein stability as demonstrated for the tetrameric water efflux channel (AqpZ) [13] and the Na*+*/H^+^ antiporter (NhaA) in *E*. *coli* [14]. Cardiolipin is characterized by four fatty acyl chains mainly unsaturated, and a small head-group. Based on cross-sectional area and volume relative to acyl chains, cardiolipin adopts a cone-shape, explaining its propensity to stabilize negative membrane curvature and to favor the transition from a lamellar, bilayer arrangement to a non-lamellar phase, inverted hexagonal (H_II_) phase upon sequestration [15;16]. These features result in the creation of localized membrane constrictions that are primed for fission and fusion [17;18], both critical for events involved in bacterial division [19].

With the aim to target lipid bacterial membranes and especially cardiolipin, we developed a relation-activity structure (RAS) program from neamine and neosamine [20–23], the primary scaffolds of aminoglycoside antibiotics. Aminoglycosides are broad-spectrum antibiotics typically used to treat Gram-negative infections and as second-line of defense treatment for multidrug-resistant (MDR) tuberculosis (*Mycobacterium tuberculosis*) [24] We identified 3’,6-dinonylneamine (3’,6-DiN Neamine) as a lead amphiphilic neamine derivative [23;25–27] (S1 Fig). On wild type *P*. *aeruginosa*, the minimal inhibitory concentration (MIC) of 3’,6-dinonylneamine is low (1–4 μg/mL) with moderate toxicity on J774 macrophages (viability 78.4% at 30 μM) [22]. 3’,6-dinonylneamine also shows bactericidal effect on a wide range of Gram-negative and Gram-positive bacteria including strains resistant to colistin [25]. In addition, 3’,6-dinonylneamine inhibits biofilm formation [25]. From a molecular point of view, we demonstrated an interaction of 3′,6-dinonylneamine with outer membrane’s lipopolysaccharides [25] and inner membrane’s anionic phospholipids mostly cardiolipin leading to membrane permeabilization and depolarization [27]. This interaction is favored by the positive-charge and inverted cone-shaped of 3’,6-dinonylneamine and the negative-charge and cone-shaped of cardiolipin [26;27]. On *P*. *aeruginosa*, 3’, 6-dinonylneamine induced morphological defects characterized by an increase in membrane curvature, a loss of rod shape morphology and a decrease of bacterial cell length [26]. We also demonstrated cardiolipin relocation and clustering as a result of exposure of *P*. *aeruginosa* to a cardiolipin-acting amphiphilic aminoglycoside antibiotic [26].

The objective of this study is to characterize the effect of cardiolipin on *P*. *aeruginosa* growth and on the antimicrobial activity of 3’,6-dinonylneamine. We image morphology of *P*. *aeruginosa* and monitor the growth rate, and the asymmetrical division. By using membrane models mimicking the *P*. *aeruginosa* membrane, we determine membrane fusion, membrane permeabilization and hydration changes induced by 3’,6-dinonylneamine upon increasing contents in cardiolipin. Our findings may contribute towards the understanding of the molecular mechanism involved in the antimicrobial activity of neamine derivatives as promising antibacterial compounds.

## Experimental procedures

3’,6-dinonylneamine was synthesized by Decout and colleagues [21;22]. 1-palmitoyl-2-oleoyl-*sn*-glycero-3-phosphoethanolamine (POPE), 1-palmitoyl-2-oleoyl-*sn*-glycero-3-phosphoglycerol (POPG), 1,3-bis-(*sn*-3’-phosphatidyl)-*sn*-glycerol (cardiolipin; CL: from *E*. *coli*) were purchased from Avanti Polar Lipids (Alabaster, AL). Octadecyl rhodamine B (R18) was purchased from Invitrogen (Paisley, Scotland, UK). Laurdan and calcein were purchased from Sigma-Aldrich. All solvents (analytical grade) were purchased from E. Merck AG.

### Large unilamellar vesicles (LUVs) preparation

Large unilamellar vesicles (LUVs) were prepared by using the extrusion [28;29] method from multilamellar vesicles (MLVs) [30]. Phospholipids POPE:POPG:CL (1 mg/mL in CHCl_3_/CH_3_OH 2:1 v/v) were mixed in the desired molar ratio. POPE and POPG lipids were used at a molar ratio of 60 and 21, respectively, and cardiolipin was added at three different ratios 0, 11, and 20. Preparation of liposomes and their characterization (size and phospholipid concentrations [31]) were done as described in detail previously [27]. Liposomes at 5 μM phospholipids were incubated for 10 min with different concentrations of 3’,6-dinonylneamine.

### Membrane fusion experiment

Membrane fusion was monitored on LUVs. Octadecyl rhodamine B (R18), a lipid-soluble probe, was incorporated into a lipid membrane at a self-quenching concentration. Hence a decrease in its surface density [27;32] is associated with an increase in the fluorescence intensity of the preparation. The experiments were performed as described previously in details [27;33] by using an LS55 spectrofluorimeter (PerkinElmer) and excitation wavelength (λ_exc_) a1nd emission wavelength (λ_em_) fixed at 560 and 590 nm, respectively. The fusion percentage was calculated according to the Eq (1)

$$\mathrm{Fusion}\;\left(\%\right)=\left(\frac{F_{t}-F_{0}}{F_{\infty }-F_{0}}\right)\;X\;100$$ (1)

where F_t_, F_0_ and F_∞_ are the fluorescence intensities measured at time t, at time 0, and after the addition of detergent (0.5% triton X-100) to disrupt the membrane of the liposomes, respectively.

### Calcein-release measurement for permeability study

The permeabilizing effect of 3’,6-dinonylneamine was investigated by using calcein-filled LUVs [27; 34]. Calcein-filled LUVs composed of POPE/POPG/CL (60:21:20) (60:21:11) and (60:21:0) were prepared using 10 mM Tris-HCl (pH 7.4) and 73 mM calcein (390 mosmol/L measured with the freezing point technique (Knauer osmometer automatic, Berlin, Germany).

Elimination of non-entrapped calcein and determination of calcein release from liposomes were performed as described in detail previously [27] by using an LS55 spectrofluorimeter (PerkinElmer) with λ_exc_ and λ_em_ fixed at 472 and 512 nm, respectively.

The amount of calcein released after time t was calculated according to Eq (2):

$$RF\;\left(\%\right)=\left(\frac{F_{t}-F_{0}}{F_{\infty }-F_{o}}\right)\;X\;100$$ (2)

where RF—fraction of calcein released, F_t_, F_0_ and F_∞_ are the fluorescence intensities measured at time t, at time o, and after the addition of Triton X-100, respectively.

### Generalized Polarization of Laurdan

Amphiphilic Laurdan fluorescent probe resides in the interfacial region of the lipid bilayer membrane. The large excited state dipole moment of Laurdan and the dipolar relaxation effect induced, explains the huge use of Laurdan to monitor the extent of hydration of lipid membrane [26;35;36]. The shift in its emission spectrum was monitored and the steady state fluorescence parameter known as Generalized Polarization (GP) was calculated. After preparation of Laurdan labeled LUVs they were diluted at a concentration of 5 μM phospholipids with a lipid to Laurdan ratio of 100:2. Laurdan was excited (LS55 Perkin Elmer) at 350 nm, and GP was calculated from the emission intensities at 440 nm (*I*_440_) and 485 nm (*I*_480_) by using Eq (3)

$$GP=\left(\frac{I_{440}-I_{485}}{I_{440}+I_{485}}\right)$$ (3)

### Bacterial strain and growth conditions

Trypticase soy agar (TSA) plates were used to grow *P*. *aeruginosa* strains ATCC 27853 at 37°C. One colony of bacteria was suspended in Cation Adjusted–Müller Hinton Broth (CaMHB) and incubated overnight at 37°C on a rotary shaker (130 rpm). For growth study, the bacterial suspension was diluted 100 fold in CaMHB and incubated (130 rpm; 37°C; 4 h) with selected concentrations of cardiolipin and/or 3’,6-dinonylneamine. Growth rate calculation was performed as described elsewhere [26].

### Scanning electron microscopy

*P*. *aeruginosa* was grown to mid log (OD: 0.3 at 600 nm). Bacteria were washed in phosphate buffered saline (0.1 M PBS, pH 7.4) and then treated with cardiolipin and/or 3’,6-dinonylneamine for 1 hour at different concentrations. A suspension of bacterial cells was immobilized on poly-L-lysine coated coverslips for 10 min at room temperature. After washing in buffer–to remove the excess of free floating bacteria- coverslips were incubated in 1% glutaraldehyde in order to cross-link the fixed bacteria on poly-lysine coating. We further post-fixed the samples in 1% osmium tetroxide in cacodylate buffer for 2 h at 4°C and washed in water to eliminate traces of remaining osmium tetroxide. Samples were then dehydrated in graded series of ethanol, critical point dried and coated with 10 nm of gold. Samples were observed in a CM12 Philips electron microscope at 80 kV with the secondary electron detector. The i-TEM imaging software was used for the analysis of bacterial length. The asymmetrical division was calculated by measuring the differences in daughter lengths from the position of cell division.

## Results

We first investigated the effect of increasing contents in cardiolipin on changes in membrane fusion, membrane permeabilization and membrane hydration induced by 3’,6-dinonylneamine by using membrane models mimicking the *Pseudomonas aeruginosa* membrane. Moving onto bacteria, we studied the potential effect of cardiolipin on morphology, growth rate and division of *P*. *aeruginosa* as well as the effect on the antimicrobial activity of a new promising antibiotic, the 3’, 6-dinonylneamine.

### Effect of cardiolipin on membrane fusion induced by 3’,6-dinonylneamine

**Fig 1:**
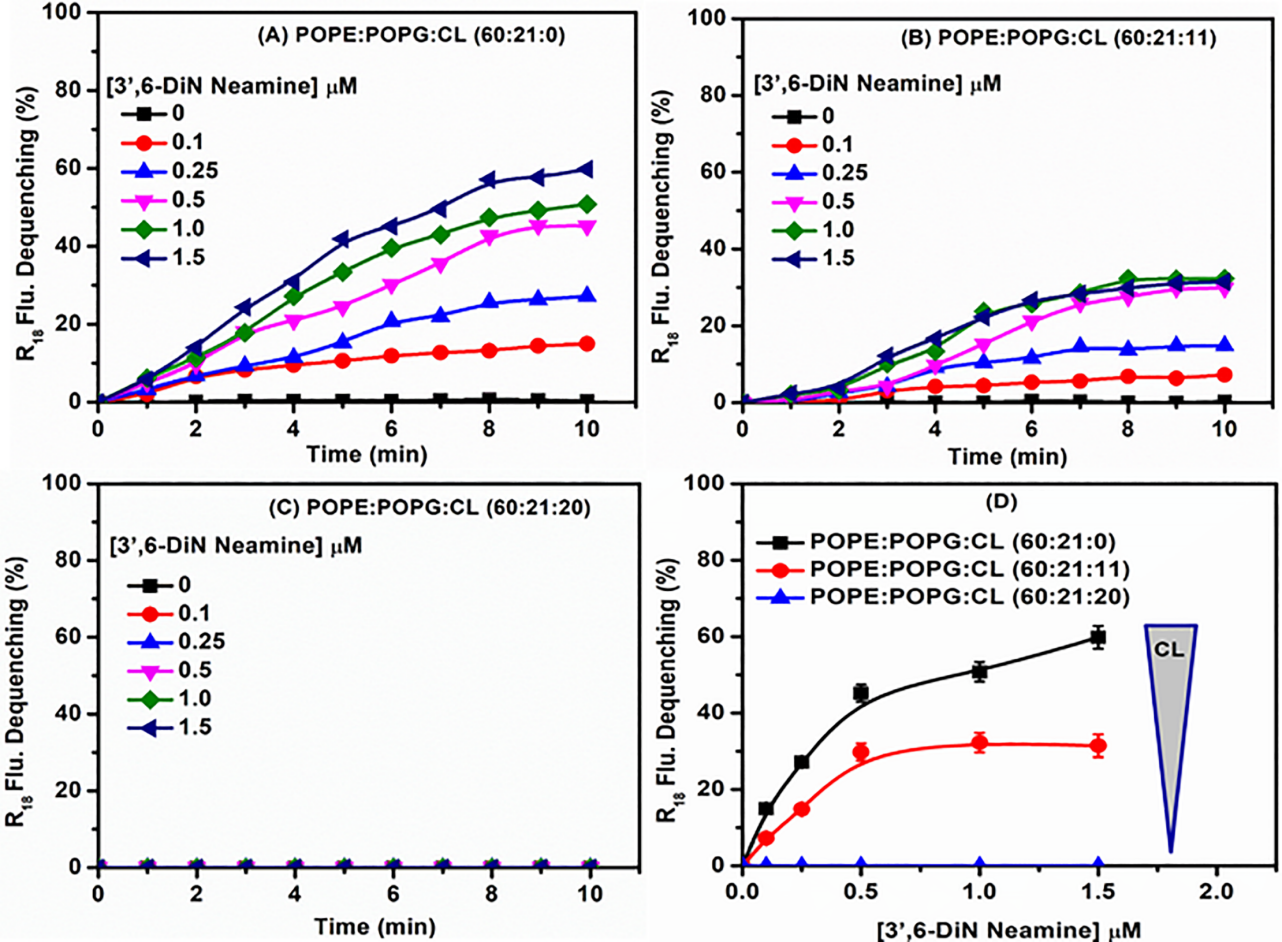
Time and concentration dependence of membrane fusion with 3’,6-dinonylneamine as assessed by R18 fluorescence dequenching. Time dependence of membrane fusion with 3’,6-dinonylneamine (A) POPE:POPG: CL (60:21:0); (B) POPE:POPG:CL (60:21:11); (C) POPE:POPG:CL (60:21:20); (D) Concentration dependence of membrane fusion induced by 3’,6-dinonylneamine for liposomes containing various contents in cardiolipin after 10 min. The data represent the mean ± SEM of three separate experiments; for sake of clarity SEM were omitted in panels A-C but were always < 4%.

To investigate whether the ability of 3’,6-dinonylneamine to induce lipid membrane fusion, a critical event in bacterial division, is dependent upon the presence of cardiolipin, we carried out experiments based on octadecyl rhodamine B (R18) fluorescence dequenching [32]. We used large unilamellar vesicles (LUVs) composed of POPE (60%) and POPG (21%) and varying amounts of cardiolipin (CL) (molar ratio 0, 11, and 20%). LUVs labeled with octadecyl rhodamine B (R18) were mixed with unlabeled LUVs and then 3’,6-dinonylneamine was added at increasing concentrations (0–1.5 μM). In the absence of cardiolipin (POPE: POPG: CL [60:21:0] LUVs) (Fig 1A), the addition of 3’,6-dinonylneamine causes a fast increase in fluorescence even at very low concentration (0.1 μM) of aminoglycoside derivative. The fusion process reached a plateau value after 8 minutes for all the concentration range of 3’,6-dinonylneamine. The effect is inversely related to contents in cardiolipin since with cardiolipin present at 11% (Fig 1B) the percentage of fusion was lower as compared to that observed with liposomes without cardiolipin (31.5% *versus* 60% at the highest 3’,6-dinonylneamine concentration, 1.5 μM). At cardiolipin content of 20% (Fig 1C), no fusion was observed. Fig 1D shows at 10 min, the percentage of membrane fusion at the three selected molar ratio of cardiolipin, with increasing concentrations of 3’,6-dinonylneamine. Membrane fusion decreases with increased ratios of cardiolipin content in despite of the concentration of the amphiphilic neamine derivative. At 20% of cardiolipin, no fusion was observed even at the highest concentration of 3’,6-dinonylneamine. At 0 and 11% molar ratio of cardiolipin, the percentage of fusion reached a plateau value (45% and 30%, respectively) at 0.5 μM of 3’,6-dinonylneamine.

### Effect of cardiolipin on membrane permeability induced by 3’,6-dinonylneamine

**Fig 2:**
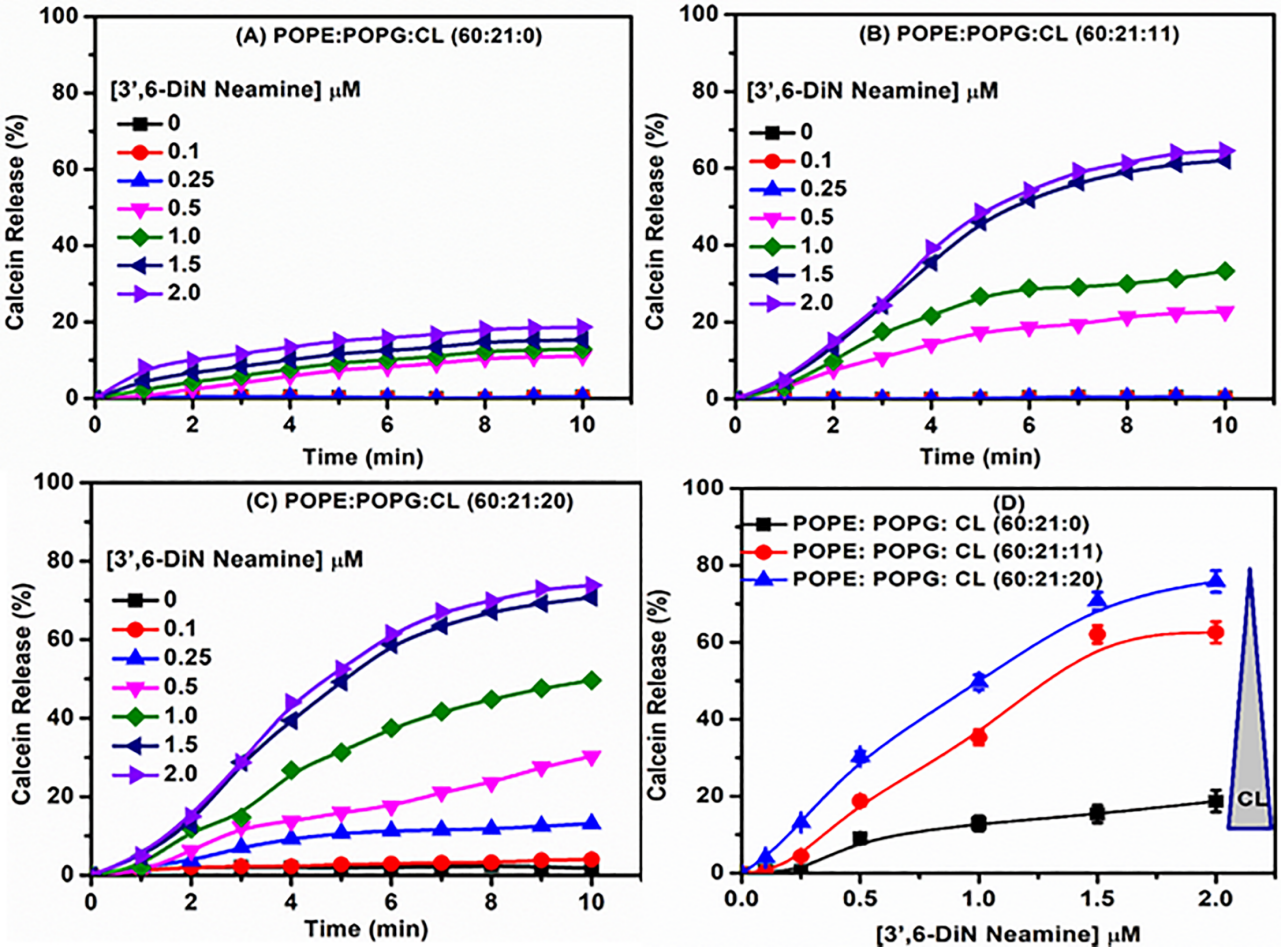
Time and concentration dependence of membrane permeability with 3’,6-dinonylneamine as assessed by calcein release from liposomes. Time dependence of membrane permeability with 3’,6-dinonylneamine (A) POPE:POPG:CL (60:21:0); (B) POPE:POPG:CL (60:21:11); (C) POPE:POPG:CL (60:21:20); (D) Concentration dependence of membrane permeability induced by 3’,6-dinonylneamine for liposomes containing various contents in cardiolipin after 10 min. The data represent the mean ± SEM of three separate experiments; for sake of clarity SEM were omitted in panels A-C but were always < 4%.

To know if cardiolipin is involved and critical for membrane permeabilization induced by 3’,6-dinonylneamine, we monitored calcein release from liposomes in which calcein was entrapped at self-quenching concentrations. Fig 2A–2C shows time-dependent calcein release induced by 3’,6-dinonylneamine, with variations of cardiolipin contents from 0–20% in POPE: POPG (60:21) LUVs. In a cardiolipin free membrane model system (Fig 2A), increased concentrations of 3’,6-dinonylneamine induced a slow increase in fluorescence intensity. After 10 min, at the highest concentration of the amphiphilic neamine derivative used (2 μM), only 10% to 15% of the encapsulated calcein was released from liposomes. When cardiolipin was inserted within liposomes, a prompt enhancement of calcein release was observed (around 62.5% and 76.0% release of the encapsulated calcein) at 11 (Fig 2B) and 20 (Fig 2C) % cardiolipin respectively. The effect of cardiolipin on membrane permeability induced by increasing concentrations of 3’,6-dinonylneamine is further illustrated in Fig 2D. A marked increase in membrane permeability correlated with increased molar ratio of cardiolipin in LUVs as observed for all concentrations of 3’,6-dinonylneamine investigated.

### Effect of cardiolipin on membrane hydration induced by 3’,6-dinonylneamine

**Fig 3:**
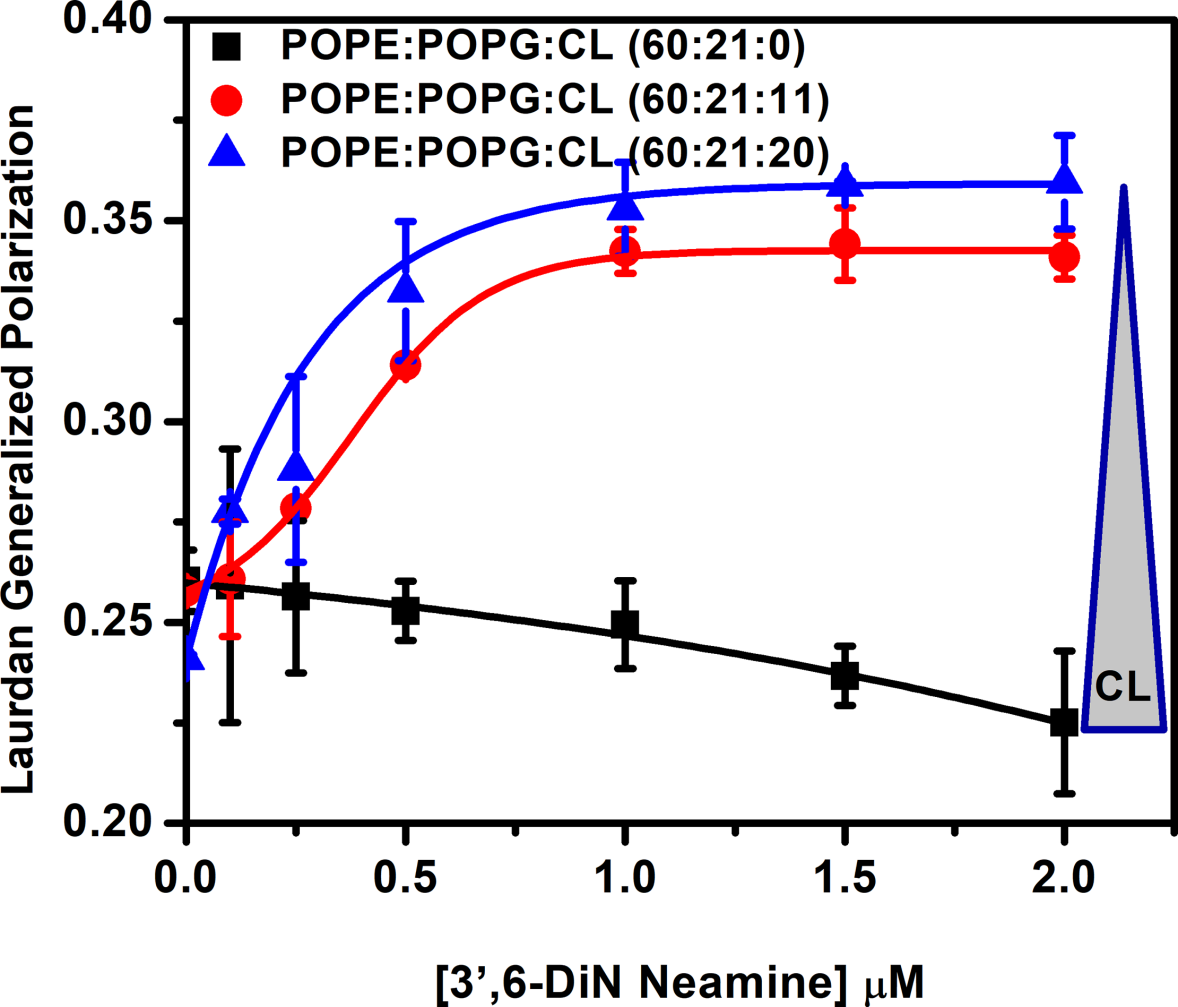
Generalized Polarization values as a function of 3’,6-dinonylneamine concentrations in LUVs (POPE:POPG) containing cardiolipin at three different molar ratio (0, 11, 20). The data represent the mean ± SEM of three separate experiments.

To further elucidate the mechanisms involved in the cardiolipin dependency changes of membrane fusion and membrane permeabilisation induced by 3’,6-dinonylneamine, we used Laurdan for monitoring membrane order and hydration. We followed changes in fluorescence intensity and calculated Generalized Polarization (GP) to establish the ability of 3’,6-dinonylneamine to modulate hydration of the lipid bilayers containing different percentages of cardiolipin. Fig 3 shows the Generalized Polarization (GP) values as a function of 3’,6-dinonylneamine concentrations. Three different liposomes (POPE:POPG vesicles) with different molar ratio of cardiolipin (0, 11, and 20%) were selected. Without cardiolipin, Generalized Polarization (GP) value decreased with increase in concentration of 3’,6-dinonylneamine whereas in presence of cardiolipin a reverse effect was observed with a plateau value reached at 1 μM of 3’,6-dinonylneamine. The Generalized Polarization (GP) value increased with increasing concentrations in 3’,6-dinonylneamine concentrations in presence of 11 and 20% cardiolipin (p = 0.66 for statistical analysis of the slope). Maximum enhancement of Generalized Polarization (GP) value was observed at 20% cardiolipin.

Together, these results obtained on lipid membrane models highlighted the critical role of cardiolipin on the effects induced by 3’,6-dinonylneamine by decreasing membrane fusion and hydration, and increasing membrane permeability of lipid membranes. The further question was to demonstrate if cardiolipin also played a key role in bacterial growth and if it could be involved in the antibacterial activity of 3’,6-dinonylneamine on *P*. *aeruginosa*.

### Effect of cardiolipin on *P*. *aeruginosa* growth and division

**Fig 4:**
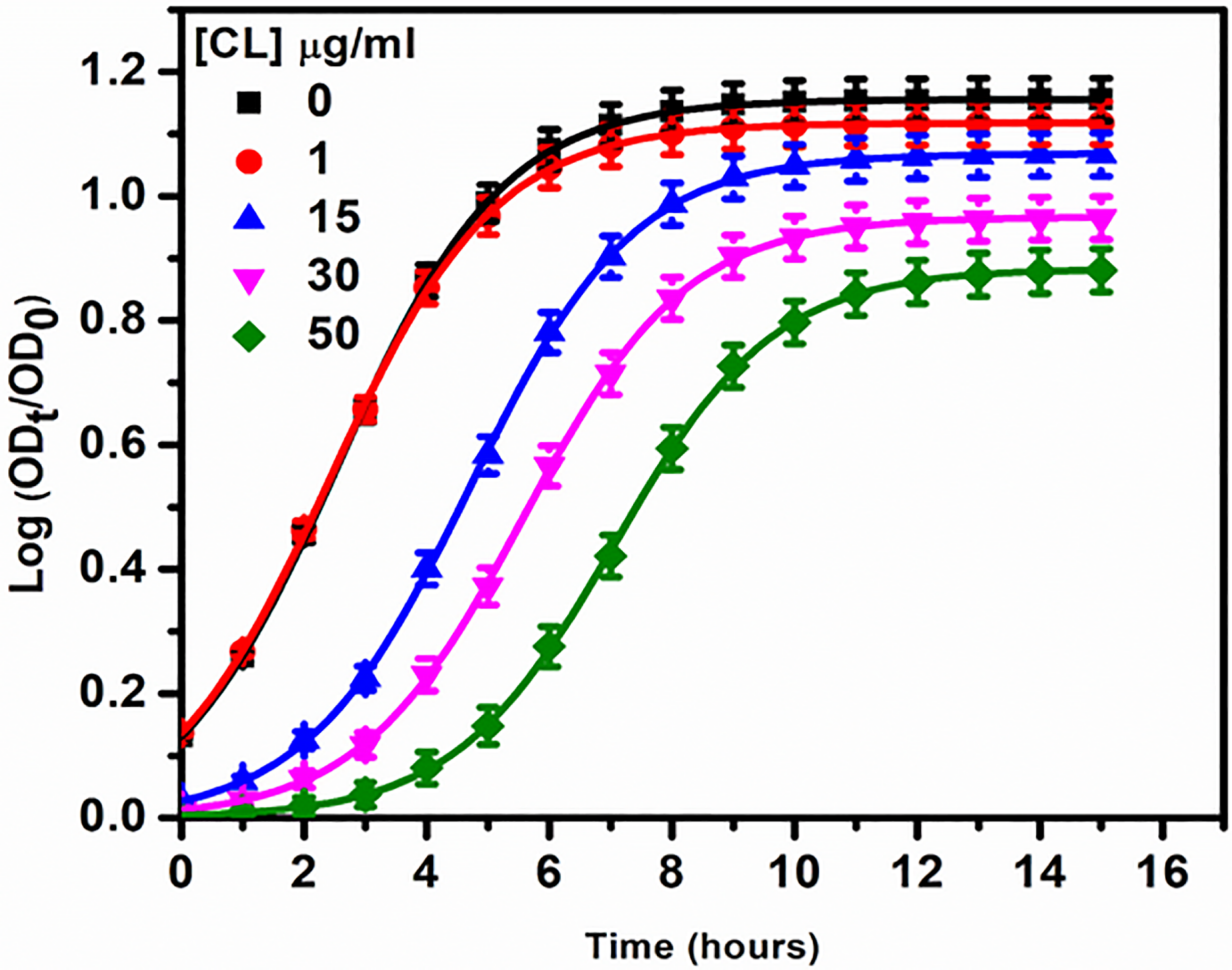
Growth curve of *P*. *aeruginosa* with increase in concentrations of cardiolipin in the growth medium. The data represent the mean ± SEM of three separate experiments.

Based on results obtained from membrane models, we wanted to investigate dependence of cardiolipin on the antimicrobial activity of 3’,6-dinonylneamine in *P*. *aeruginosa* (ATCC 27853). We first monitored the growth rate of *P*. *aeruginosa* in the presence of increasing concentrations of cardiolipin in the growth medium (Fig 4). At 1 μg/ml of cardiolipin, no effect on *P*. *aeruginosa* growth was observed. Above this concentration, cardiolipin significantly affected the growth curve of *P*. *aeruginosa* by delaying the time required to observe 50% of the maximal effect and by decreasing the maximal value of log (OD_t_/OD_0_).

**Fig 5:**
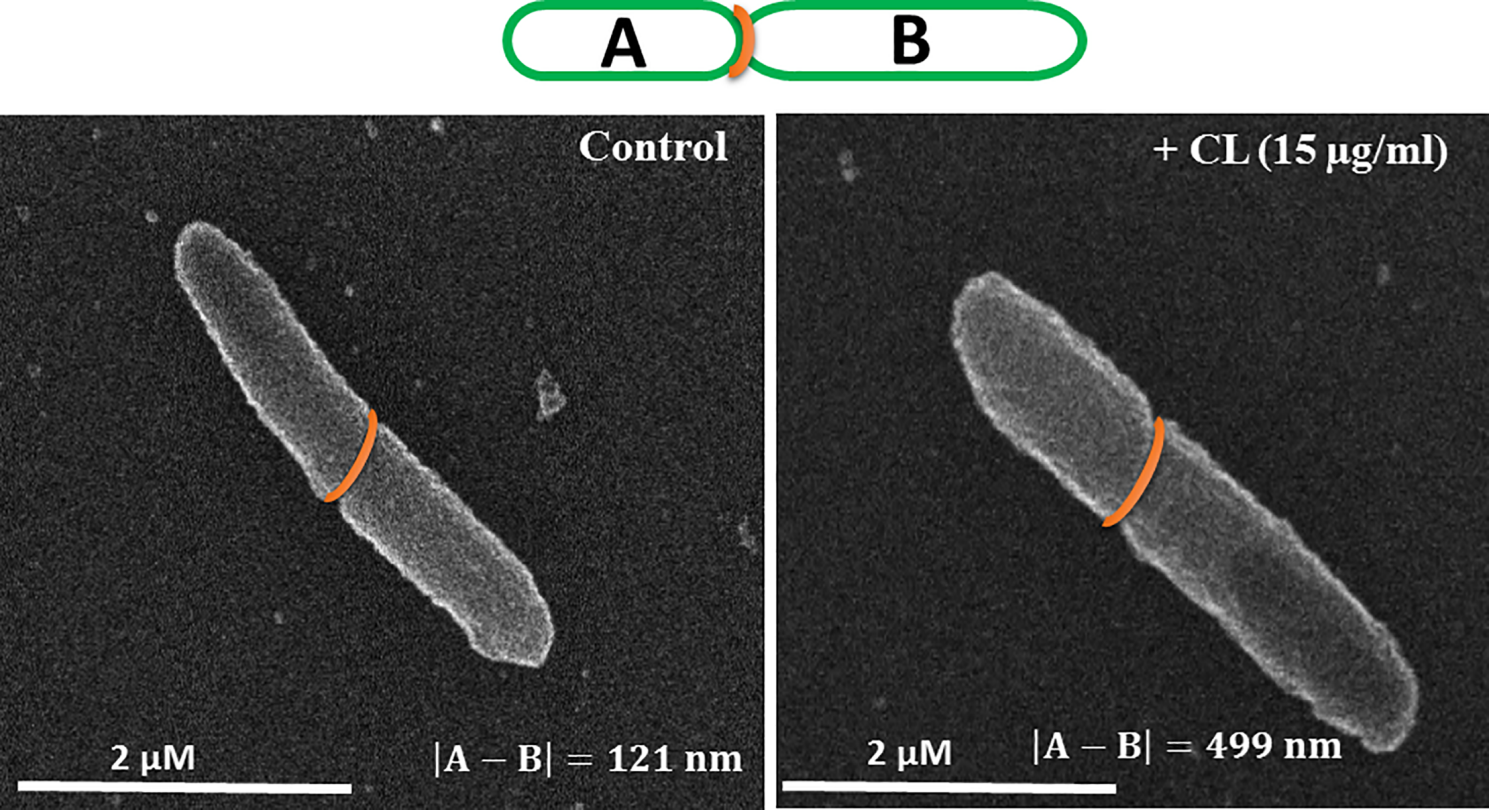
Scanning electron microscopy representative images of *P*. *aeruginosa* without or with cardiolipin added in the growth medium.

Interestingly, by using scanning electron microscopy (Fig 5), we showed that the presence of cardiolipin in the growth medium (15 μg/ml) induced asymmetric growth of bacteria. This effect was not dependent upon the presence of the amphiphilic neamine derivative.

### Effect of cardiolipin on growth of *P*. *aeruginosa* in the presence or absence of 3’,6-dinonylneamine

**Fig 6:**
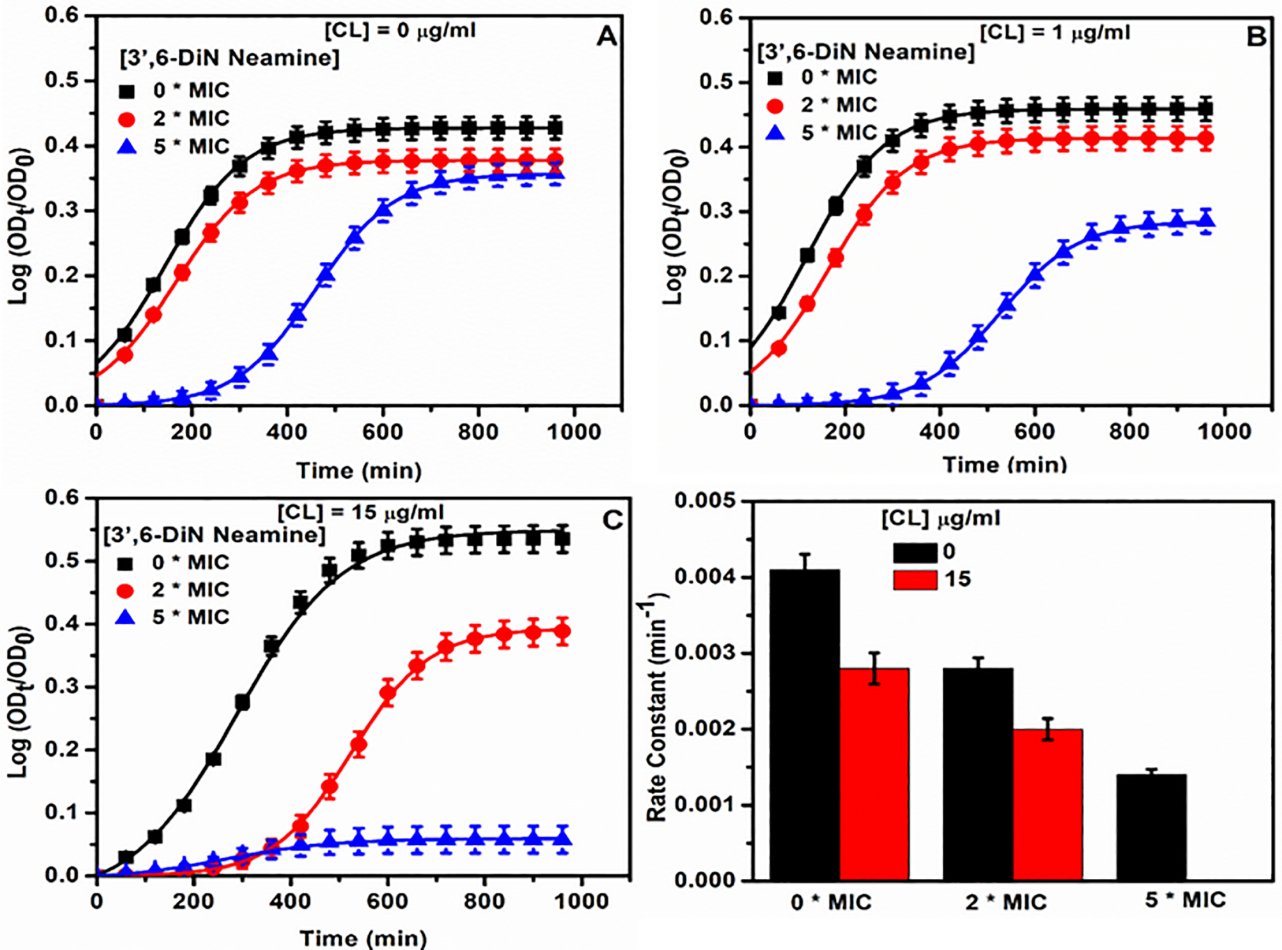
Time and concentration dependence of *P*. *aeruginosa* growth curve with increased concentrations of 3',6-dinonylneamine (0, 2, and 5 times MIC) in the growth medium containing or not cardiolipin. (A) 0 μg/ml of cardiolipin, (B) 1 μg/ml of cardiolipin, and (C) 15 μg/ml of cardiolipin. (D) Histograms for growth rate constant (min^-1^) in the absence or presence of cardiolipin (15 μg/ml) with 3',6-dinonylneamine (0, 2 and 5 times MIC). The data represent the mean ± SEM of three separate experiments.

The minimum inhibitory concentration of 3’,6-dinonylneamine against *P*. *aeruginosa* is 1–4 μg/ml (23;25–27). When cardiolipin was added to the medium (1 and 15 μg/ml) (Fig 6B and 6C), the growth curves of *P*. *aeruginosa* in presence of increasing amounts of 3’,6-dinonylneamine were monitored in comparison with data obtained in absence of added cardiolipin (Fig 6A). The bacterial growth was delayed and the growth rate decreased with increase in concentrations of 3’,6-dinonylneamine with or without cardiolipin. When cardiolipin was replaced by POPG (15 μg/ml), the bacterial growth in presence of 3’,6-dinonylneamine was only slightly decreased and delayed (S2 Fig). To highlight the effect of cardiolipin on 3’,6-dinonylneamine activity, the *P*. *aeruginosa* growth rate upon addition of cardiolipin (15 μg/ml) and 3’,6-dinonylneamine (0–2 and 5 times MIC) is illustrated in Fig 6D.

### Imaging of the effect of cardiolipin and/or 3’,6-dinonylneamine on *P*. *aeruginosa* growth

**Fig 7:**
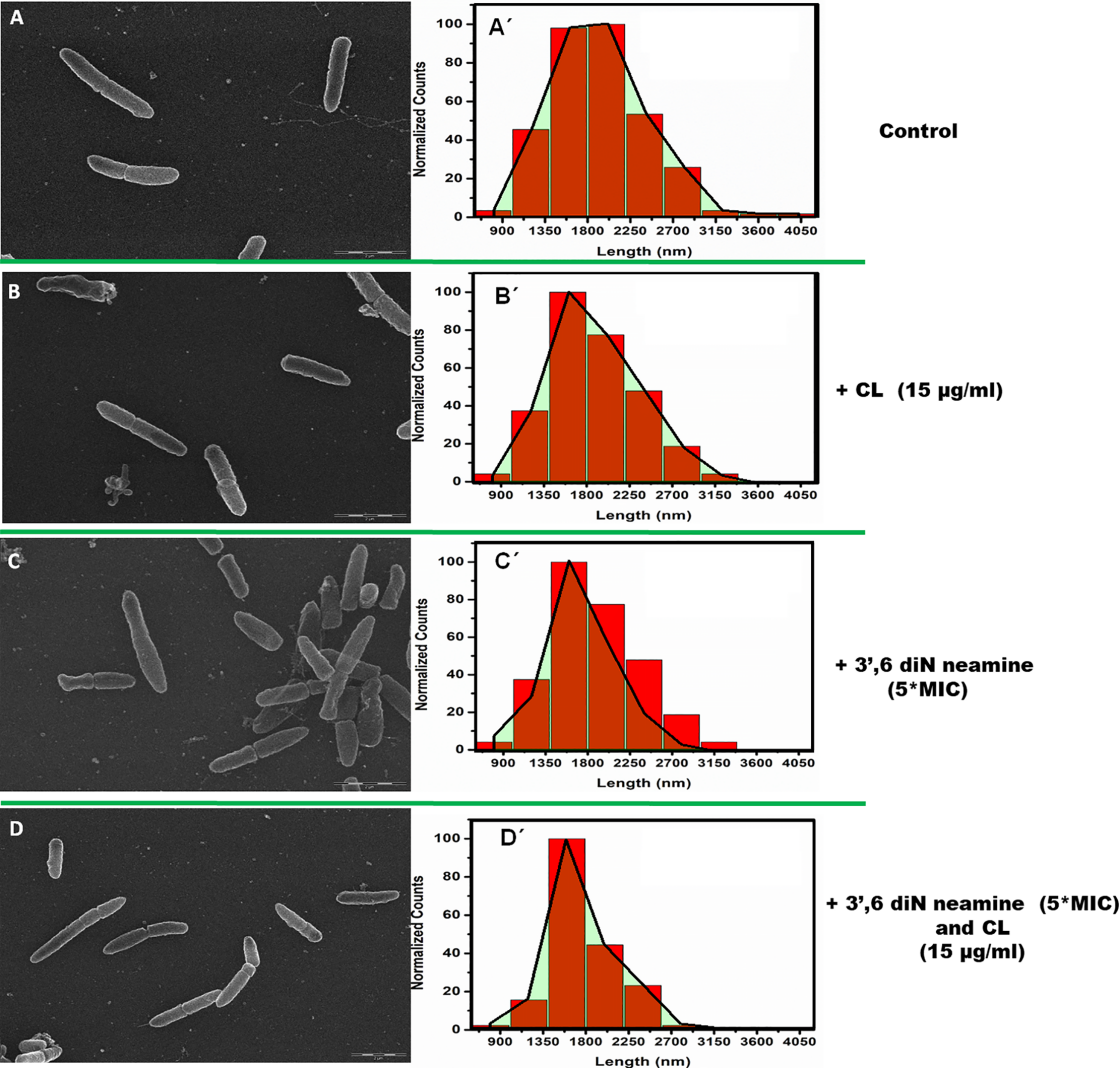
**Scanning electron microscopy images and length distribution of *P*. *aeruginosa*** incubated without (A) or in presence of cardiolipin at 15 μg/ml in the medium before sample preparation (B), 5 *MIC 3',6-dinonylneamine (C) and with both cardiolipin at 15 μg/ml and 5 *MIC 3',6-dinonylneamine (D) together before sample preparation. Mean bacterial length was 1915 nm, 1877 nm, 1649 nm and 1767 nm, respectively with SD = 35 nm. Distribution are shown, overlaid onto the distribution profile (in red). Time of incubation was 1 hour and at least 200 bacteria were monitored. Scale bars correspond to 2 μm.

In order to visualize potential effect of cardiolipin, 3',6-dinonylneamine and both compounds on growth of *P*. *aeruginosa*, we used scanning electron microscopy (Fig 7A–7D).

We normalized the number length found (which is represents Counts) in each range (range divided in 400 nm interval from 600 nm to 4000 nm) with maximum number of length in a range (Fig 7A’–7D’). Total number of length (or counts) is approximately 200. As compared to control (Fig 7A’), the three main features are (i) a decrease in normalized counts in the range 1800–2200 nm, (ii) a shift in maximum counts from 1800–2200 nm to 1400–1800 range from control to conditions where *P*. *aeruginosa* was incubated with cardiolipin (Fig 7B’) and 3’,6-dinonyl neamine (Fig 7C’) or both compounds (Fig 7D’), and (iii) a decrease of the size heterogeneity. These effects were observed in presence of cardiolipin (Fig 7B’), 3’,6-dinonyl neamine (Fig 7C’), and the mixture of both (Fig 7D’) with an increased in their extent (S3 Fig).

## Discussion

Binding of antibiotics at the septum of dividing Gram-positive [37;38] and Gram-negative [19] bacteria where cardiolipin is enriched [39] could be a unique target in order to design new antibacterial molecules. Interactions with bacterial cell membrane at discrete domains [26;40], could result in the dispersion of these domains and often in the disruption of functions governed by those domains [4;40–42]. This is exactly what we demonstrated on the life-threatening Gram-negative bacteria, *P*. *aeruginosa* with the amphiphilic neamine derivative, 3’,6-dinonylneamine [26] even the mechanisms are still unclear, requiring further study.

Increasing contents in cardiolipin in growth medium delayed *P*. *aeruginosa* growth and favored the asymmetric growth. Heterogeneity in bacterial length arises because of the unusual, unipolar nature in growth. In presence of cardiolipin, the division of the asymmetrically growing single mother cell gives rise to a number of daughter cells that differ in size [43]. This probably constitutes a physiological response of bacteria to stress since asymmetric division generates distinct cell types that may help bacteria to exploit patchy and variable, environments more effectively [44]. Interestingly, in the same time, the antimicrobial activity of 3’,6-dinonylneamine, an amphiphilic aminoglycoside which binds to and requires cardiolipin for its proper activity, increased with increasing contents in cardiolipin. This effect is in agreement with that reported by Molohon and coll [37] who have shown that exogeneous cardiolipin increases sensitivity to plantazolicin, a linear azole-containing peptide active against the Gram-positive *Bacillus anthracis*. Cardiolipin could increase bacterial respiration resulting in potentialisation of the killing effect of bactericidal antibiotics [45]. However, the mechanism is probably more complex and the metabolic state of bacteria could impact antibiotic efficacy. In this line, daptomycin exhibits an antagonistic relationship with cardiolipin in Gram-positive *Enterococci* [46]. The role of cardiolipin on the activity of membranes targeting antibiotics is probably more complex than it seems at first sight. Several non-exclusive processes could be involved including (i) potential effect on cardiolipine synthases, (ii) changes in location of cardiolipin from the inner to outer membranes and (iii) equilibrium between the antibiotic and cardiolipin. In addition, the cross-talk between PG and cardiolipin metabolism [47] could be critical.

Based on our previous studies on *P*. *aeruginosa* [26], we suggest that amphiphilic aminoglycoside derivatives recruit cardiolipin in regions of high negative curvature leading to changes in its location which can in turn result in inhibition of membrane scission through changes of the topology of proteins involved in in cell division (FtsZ, FtsA) and cell shape regulation (MreB). Indeed, 3’,6-dinonylneamine unsettled rod shape regulation protein mCherry-MreB and inhibited L-spherosplasts to restore their initial rod shape [26]. In agreement with results on lipid models, interaction between 3’,6-dinonylneamine and cardiolipin could result in decreasing line tension and/or the discontinuity in elastic properties between the cardiolipin-rich and cardiolipin-poor phases which are known to spontaneously induce fission. Alternatively, binding of amphiphilic neamine derivatives to cardiolipin could trigger a conformational change in FtsZB, ultimately decreasing protein activity. Thus, FtsZ and MreB functions would be fine-tuned through modulation of cardiolipin levels.

Additional insight on the effect of cardiolipin on the antibacterial activity of amphiphilic aminoglycoside derivatives results from studies performed on membrane models mimicking bacterial membranes of *P*. *aeruginosa*. We demonstrated that cardiolipin increased membrane permeability and decreased hydration (as assessed by increase of calcein release and increase of Generalized Polarization (GP) of Laurdan). In the absence of cardiolipin, the Generalized Polarization (GP) value slightly decreased with an increase in 3’,6-dinonylneamine concentrations suggesting an increase in inter backbone distance between POPE and POPG and penetration of water molecules into the bilayer. At the opposite, in presence of cardiolipin, 3’,6-dinonylneamine induced a huge increase in the Generalized Polarization GP, reflecting a decrease of hydration likely due to interaction of 3’,6-dinonylneamine with cardiolipin. This could explain enhanced pore formation. Decrease of membrane hydration upon increasing cardiolipin contents could also explain the decreased hemi-fusion process induced by 3’,6-dinonylneamine as evidenced by measuring fluorescence dequenching of octadecyl rhodamine B (R18) upon increasing contents in cardiolipin. One potential molecular mechanism could result from the complementary inverted cone shaped of 3’,6-dinonylneamine and cone-shaped of cardiolipin preventing hexagonal phase formation and decreasing fusion process.

Expanding on these studies will provide insights into how increase in cardiolipin contents, related to emergence of resistance, could influence antibiotic efficiency and modulate protein-protein interactions [48].

## Conclusion

We evidenced cardiolipin-dependency on antimicrobial activity of 3’,6-dinonylneamine. Cardiolipin is responsible for decrease in growth rate, asymmetric growth defects and enhanced antimicrobial activity of 3’,6-dinonylneamine. Upon increase in cardiolipin contents in membrane models mimicking membranes of *P*. *aeruginosa*, 3’,6-dinonylneamine induced increase in membrane permeabilization and decreased in membrane hydration probably related with inhibition of membrane fusion. We anticipate that these findings will be influential for understanding the role of lipids in modulating functions of proteins or drug activity.

## Supporting information

S1 Fig Chemical structures of 3’,6-dinonylneamine and cardiolipin. (DOCX)

S2 Fig Length distribution of *P*. *aeruginosa* in the size range of 1800–2200 nm as analyzed from scanning electron microscopy images. *P*. *aeruginosa* were incubated (1 h) in presence of cardiolipin at 15 μg/ml in the medium before sample preparation, 5 *MIC 3',6-dinonylneamine and with both cardiolipin at 15 μg/ml and 5 *MIC 3',6-dinonylneamine together.
(DOCX)

S3 Fig Time and concentration dependence of *P*. *aeruginosa* growth curve with increased concentrations of 3',6-dinonylneamine (0, 2 and 5 times MIC) in the growth medium cardiolipin or POPG. (DOCX)

## References

[1] Hughes JS, Hurford A, Finley RL, Patrick DM, Wu J, Morris AM. How to measure the impacts of antibiotic resistance and antibiotic development on empiric therapy: new composite indices. BMJ Open 2016; 6(12):e012040 10.1136/bmjopen-2016-012040 27986734 PMC5168677

[2] Ghosh C, Haldar J. Membrane-Active Small Molecules: Designs Inspired by Antimicrobial Peptides. ChemMedChem 2015; 10(10):1606–1624. 10.1002/cmdc.201500299 26386345

[3] Mingeot-Leclercq MP, Decout JL. Bacterial lipid membranes as promising targets to fight antimicrobial resistance, molecular foundations and illustration through the renewal of aminoglycoside antibiotics and emergence of amphiphilic aminoglycosides. Med Chem Commun 2016; 7:586–611.

[4] Epand RM, Epand RF. Lipid domains in bacterial membranes and the action of antimicrobial agents. Biochim Biophys Acta 2009; 1788(1):289–294. 10.1016/j.bbamem.2008.08.023 18822270

[5] Lopez GA, Heredia RM, Boeris PS, Lucchesi GI. Content of cardiolipin of the membrane and sensitivity to cationic surfactants in Pseudomonas putida. J Appl Microbiol 2016; 121(4):1004–1014. 10.1111/jam.13238 27442261

[6] Arias-Cartin R, Grimaldi S, Pommier J, Lanciano P, Schaefer C, Arnoux P et al Cardiolipin-based respiratory complex activation in bacteria. Proc Natl Acad Sci U S A 2011; 108(19):7781–7786. 10.1073/pnas.1010427108 21518899 PMC3093509

[7] Lewis RN, McElhaney RN. The physicochemical properties of cardiolipin bilayers and cardiolipin-containing lipid membranes. Biochim Biophys Acta 2009; 1788(10):2069–2079. 10.1016/j.bbamem.2009.03.014 19328771

[8] Romantsov T, Guan Z, Wood JM. Cardiolipin and the osmotic stress responses of bacteria. Biochim Biophys Acta 2009; 1788(10):2092–2100. 10.1016/j.bbamem.2009.06.010 19539601 PMC3622477

[9] Luevano-Martinez LA, Kowaltowski AJ. Phosphatidylglycerol-derived phospholipids have a universal, domain-crossing role in stress responses. Arch Biochem Biophys 2015; 585:90–97. 10.1016/j.abb.2015.09.015 26391924

[10] Keller R, Arioz C, Hansmeier N, Stenberg-Bruzell F, Burstedt M, Vikstrom D et al The Escherichia coli Envelope Stress Sensor CpxA Responds to Changes in Lipid Bilayer Properties. Biochemistry 2015; 54(23):3670–3676. 10.1021/acs.biochem.5b00242 25993101

[11] Mukhopadhyay R, Huang KC, Wingreen NS. Lipid localization in bacterial cells through curvature-mediated microphase separation. Biophys J 2008; 95(3):1034–1049. 10.1529/biophysj.107.126920 18390605 PMC2479595

[12] Oliver PM, Crooks JA, Leidl M, Yoon EJ, Saghatelian A, Weibel DB. Localization of anionic phospholipids in Escherichia coli cells. J Bacteriol 2014; 196(19):3386–3398. 10.1128/JB.01877-14 25002539 PMC4187673

[13] Laganowsky A, Reading E, Allison TM, Ulmschneider MB, Degiacomi MT, Baldwin AJ et al Membrane proteins bind lipids selectively to modulate their structure and function. Nature 2014; 510(7503):172–175. 10.1038/nature13419 24899312 PMC4087533

[14] Gupta K, Donlan JAC, Hopper JTS, Uzdavinys P, Landreh M, Struwe WB et al The role of interfacial lipids in stabilizing membrane protein oligomers. Nature 2017; 541(7637):421–424. 10.1038/nature20820 28077870 PMC5501331

[15] Verkleij AJ, van Echteld CJ, Gerritsen WJ, Cullis PR, de Kruijff B. The lipidic particle as an intermediate structure in membrane fusion processes and bilayer to hexagonal HII transitions. Biochim Biophys Acta 1980; 600(3):620–624. 7407134 10.1016/0005-2736(80)90465-4

[16] Alessandrini A, Muscatello U. AFM and FTIR spectroscopy investigation of the inverted hexagonal phase of cardiolipin. J Phys Chem B 2009; 113(11):3437–3444. 10.1021/jp809705d 19243109

[17] Siegel DP. Inverted micellar intermediates and the transitions between lamellar, cubic, and inverted hexagonal lipid phases. I. Mechanism of the L alpha—-HII phase transitions. Biophys J 1986; 49(6):1155–1170. 10.1016/S0006-3495(86)83744-4 3719074 PMC1329699

[18] Marrink SJ, Mark AE. Molecular view of hexagonal phase formation in phospholipid membranes. Biophys J 2004; 87(6):3894–3900. 10.1529/biophysj.104.048710 15377528 PMC1304900

[19] Zweytick D, Japelj B, Mileykovskaya E, Zorko M, Dowhan W, Blondelle SE et al N-acylated peptides derived from human lactoferricin perturb organization of cardiolipin and phosphatidylethanolamine in cell membranes and induce defects in Escherichia coli cell division. PLoS One 2014; 9(3):e90228 10.1371/journal.pone.0090228 24595074 PMC3940911

[20] Baussanne I, Bussiere A, Halder S, Ganem-Elbaz C, Ouberai M, Riou M et al Synthesis and antimicrobial evaluation of amphiphilic neamine derivatives. J Med Chem 2010; 53(1):119–127. 10.1021/jm900615h 20000576

[21] Jackowski O, Bussière A, Vanhaverbeke C, Baussanne I, Peyrin E, Mingeot-Leclercq MP et al Major increases of the reactivity and selectivity in aminoglycoside O-alkylation due to the presence of fluoride ions. Tetrahedron 2012; 68:737–746.

[22] Zimmermann L, Bussiere A, Ouberai M, Baussanne I, Jolivalt C, Mingeot-Leclercq MP et al Tuning the antibacterial activity of amphiphilic neamine derivatives and comparison to paromamine homologues. J Med Chem 2013; 56(19):7691–7705. 10.1021/jm401148j 24083676

[23] Zimmermann L, Das I, Desire J, Sautrey G, Barros RS, V, El Khoury M et al New Broad-Spectrum Antibacterial Amphiphilic Aminoglycosides Active against Resistant Bacteria: From Neamine Derivatives to Smaller Neosamine Analogues. J Med Chem 2016; 59(20):9350–9369. 10.1021/acs.jmedchem.6b00818 27690420

[24] Green KD, Garneau-Tsodikova S. Resistance in tuberculosis: what do we know and where can we go? Front Microbiol 2013; 4:208 10.3389/fmicb.2013.00208 23888158 PMC3719028

[25] Sautrey G, Zimmermann L, Deleu M, Delbar A, Souza ML, Jeannot K et al New amphiphilic neamine derivatives active against resistant Pseudomonas aeruginosa and their interactions with lipopolysaccharides. Antimicrob Agents Chemother 2014; 58(8):4420–4430. 10.1128/AAC.02536-13 24867965 PMC4136028

[26] El Khoury M, Swain J, Sautrey G, Zimmermann L, Van Der SP, Decout JL et al Targeting Bacterial Cardiolipin Enriched Microdomains: An Antimicrobial Strategy Used by Amphiphilic Aminoglycoside Antibiotics. Sci Rep 2017; 7(1):10697 10.1038/s41598-017-10543-3 28878347 PMC5587548

[27] Sautrey G, El Khoury M, Dos Santos AG, Zimmermann L, Deleu M, Lins L et al Negatively Charged Lipids as a Potential Target for New Amphiphilic Aminoglycoside Antibiotics: A BIOPHYSICAL STUDY. J Biol Chem 2016; 291(26):13864–13874. 10.1074/jbc.M115.665364 27189936 PMC4919468

[28] Hope MJ, Bally MB, Webb G, Cullis PR. Production of large unilamellar vesicles by a rapid extrusion procedure: characterization of size distribution, trapped volume and ability to maintain a membrane potential. Biochim Biophys Acta. 1985; 812(1):55–65. 23008845 10.1016/0005-2736(85)90521-8

[29] Mui B1, Chow L, Hope MJ. Extrusion technique to generate liposomes of defined size. Methods Enzymol. 2003; 367:3–14. 10.1016/S0076-6879(03)67001-1 14611054

[30] Szoka F Jr, Papahadjopoulos D. Comparative properties and methods of preparation of lipid vesicles (liposomes). Annu Rev Biophys Bioeng. 1980; 9:467–508 10.1146/annurev.bb.09.060180.002343 6994593

[31] BARTLETT GR. Phosphorus assay in column chromatography. J Biol Chem 1959; 234(3):466–468. 13641241

[32] Hoekstra D, de Boer T, Klappe K, Wilschut J. Fluorescence method for measuring the kinetics of fusion between biological membranes. Biochemistry 1984; 23(24):5675–5681. 6098295 10.1021/bi00319a002

[33] Arnhold J, Wiegel D, Hussler O, Arnold K. Quenching and dequenching of octadecyl Rhodamine B chloride fluorescence in Ca(2+)-induced fusion of phosphatidylserine vesicles: effects of poly(ethylene glycol). Biochim Biophys Acta 1994; 1191(2):375–383. 8172923 10.1016/0005-2736(94)90189-9

[34] Weinstein JN, Yoshikami S, Henkart P, Blumenthal R, Hagins WA. Liposome-cell interaction: transfer and intracellular release of a trapped fluorescent marker. Science 1977; 195(4277):489–492. 835007 10.1126/science.835007

[35] Parasassi T, De Stasio G, Ravagnan G, Rusch RM, Gratton E. Quantitation of lipid phases in phospholipid vesicles by the generalized polarization of Laurdan fluorescence. Biophys J 1991; 60(1):179–189. 10.1016/S0006-3495(91)82041-0 1883937 PMC1260049

[36] Parasassi T, Gratton E. Membrane Lipid Domains and Dynamics as Detected by Laurdan Fluorescence. J Fluoresc 1995; 5:59–69. 10.1007/BF00718783 24226612

[37] Molohon KJ, Blair PM, Park S, Doroghazi JR, Maxson T, Hershfield JR et al Plantazolicin is an ultra-narrow spectrum antibiotic that targets the Bacillus anthracis membrane. ACS Infect Dis 2016; 2(3):207–220. 10.1021/acsinfecdis.5b00115 27152321 PMC4852872

[38] Garcia-Fernandez E, Koch G, Wagner RM, Fekete A, Stengel ST, Schneider J et al Membrane Microdomain Disassembly Inhibits MRSA Antibiotic Resistance. Cell 2017; 171(6):1354–1367. 10.1016/j.cell.2017.10.012 29103614 PMC5720476

[39] Mileykovskaya E, Dowhan W. Cardiolipin membrane domains in prokaryotes and eukaryotes. Biochim Biophys Acta 2009; 1788(10):2084–2091. 10.1016/j.bbamem.2009.04.003 19371718 PMC2757463

[40] Epand RM, Walker C, Epand RF, Magarvey NA. Molecular mechanisms of membrane targeting antibiotics. Biochim Biophys Acta 2016; 1858(5):980–987. 10.1016/j.bbamem.2015.10.018 26514603

[41] Johnston CW, Skinnider MA, Dejong CA, Rees PN, Chen GM, Walker CG et al Assembly and clustering of natural antibiotics guides target identification. Nat Chem Biol 2016; 12(4):233–239. 10.1038/nchembio.2018 26829473

[42] Lin TY, Weibel DB. Organization and function of anionic phospholipids in bacteria. Appl Microbiol Biotechnol 2016; 100(10):4255–4267. 10.1007/s00253-016-7468-x 27026177

[43] Christen M, Kulasekara HD, Christen B, Kulasekara BR, Hoffman LR, Miller SI. Asymmetrical distribution of the second messenger c-di-GMP upon bacterial cell division. Science 2010; 328(5983):1295–1297. 10.1126/science.1188658 20522779 PMC3906730

[44] Kysela DT, Brown PJ, Huang KC, Brun YV. Biological consequences and advantages of asymmetric bacterial growth. Annu Rev Microbiol 2013; 67:417–435. 10.1146/annurev-micro-092412-155622 23808335 PMC4001765

[45] Lobritz MA, Belenky P, Porter CB, Gutierrez A, Yang JH, Schwarz EG et al Antibiotic efficacy is linked to bacterial cellular respiration. Proc Natl Acad Sci U S A 2015; 112(27):8173–8180. 10.1073/pnas.1509743112 26100898 PMC4500273

[46] Zhang T, Muraih JK, Tishbi N, Herskowitz J, Victor RL, Silverman J et al Cardiolipin prevents membrane translocation and permeabilization by daptomycin. J Biol Chem 2014; 289(17):11584–11591. 10.1074/jbc.M114.554444 24616102 PMC4002069

[47] Khalifat N, Rahimi M, Bitbol AF, Seigneuret M, Fournier JB, Puff N, Arroyo M, Angelova MI. Interplay of packing and flip-flop in local bilayer deformation. How phosphatidylglycerol could rescue mitochondrial function in a cardiolipin-deficient yeast mutant. Biophys J. 2014; 107: 879–890. 10.1016/j.bpj.2014.07.015 25140423 PMC4142233

[48] Cong X, Liu Y, Liu W, Liang X, Laganowsky A. Allosteric modulation of protein-protein interactions by individual lipid binding events. Nat Commun 2017; 8(1):2203 10.1038/s41467-017-02397-0 29259178 PMC5736629

