# Supplementary material for: Effect of cardiolipin on the antimicrobial activity of a new amphiphilic aminoglycoside derivative on Pseudomonas aeruginosa
Source: PLoS One. 2018 Aug 20;13(8):e0201752. doi: 10.1371/journal.pone.0201752 (PMC6101366; doi:10.1371/journal.pone.0201752)
Supplement: S1 Fig — (DOCX) [file pone.0201752.s001.docx]

**SUPPORTING INFORMATION**

**S1 Fig.** **Chemical structure of 3,6-dinonyl neamine (3,6-DiNn):**

**Cardiolipin**: R1,R2,R3 and R4= fatty acid hydrophobic chains
