# Supplementary material for: Effect of cardiolipin on the antimicrobial activity of a new amphiphilic aminoglycoside derivative on Pseudomonas aeruginosa
Source: PLoS One. 2018 Aug 20;13(8):e0201752. doi: 10.1371/journal.pone.0201752 (PMC6101366; doi:10.1371/journal.pone.0201752)
Supplement: S2 Fig — P. aeruginosa were incubated (1 h) in presence of cardiolipin at 15 μg/ml in the medium before sample preparation, 5 *MIC 3',6-dinonylneamine and with both cardiolipin at 15 μg/ml and 5 *MIC 3',6-dinonylneamine together. (DOCX) [file pone.0201752.s002.docx]

**SUPPORTING INFORMATION**

**S2 Fig**

| Cardiolipin | POPG |
| --- | --- |
|  | |
| **S2 Fig. Time and concentration dependence of *P. aeruginosa* growth curve** **with increased concentrations of 3',6-dinonylneamine (0, 2 and 5 times MIC) in the growth medium cardiolipin (left) or POPG (right)**. (A) 0 µg/ml of CL or POPG, (B) 1 µg/ml of CL or POPG, and (C) 15 µg/ml of CL or POPG. Results are expressed as log (OD_t_/OD_0_) where OD_t_ was optical density at different time and OD_0_ was optical density at time zero. The data represent the mean ± SEM of three separate experiments. | |
