# Supplementary material for: Effect of cardiolipin on the antimicrobial activity of a new amphiphilic aminoglycoside derivative on Pseudomonas aeruginosa
Source: PLoS One. 2018 Aug 20;13(8):e0201752. doi: 10.1371/journal.pone.0201752 (PMC6101366; doi:10.1371/journal.pone.0201752)
Supplement: S3 Fig — (DOCX) [file pone.0201752.s003.docx]

**SUPPORTING INFORMATION**

**S3 Fig**





**S3 Fig. Length distribution of *P. aeruginosa* in the size range of 1800-2200 nm as analyzed from Scanning electron microscopy images**. *P. aeruginosa* were incubated (1 hour) in presence of cardiolipin at 15 µg/ml in the medium before sample preparation, 5 *MIC 3',6-dinonylneamine and with both cardiolipin at 15 µg/ml and 5 *MIC 3',6-dinonylneamine together. At least 200 bacteria were monitored.
